# Multimodal strategy to rescue the brain in mild cognitive impairment: Ketogenic oral nutrition supplementation with B vitamins and aerobic exercise

**DOI:** 10.1111/eci.13806

**Published:** 2022-05-12

**Authors:** Stephen C. Cunnane, Russell H. Swerdlow, Marco Inzitari, Gloria Olaso‐Gonzalez, José Viña

**Affiliations:** ^1^ Research Center on Aging and Department of Medicine Université de Sherbrooke Sherbrooke Quebec Canada; ^2^ University of Kansas Alzheimer's Disease Center, KUMC Neurodegenerative Disorders Program University of Kansas School of Medicine Lawrence Kansas USA; ^3^ REFiT Barcelona Research Group Parc Sanitari Pere Virgili and Vall d'Hebrón Institute of Research Barcelona Spain; ^4^ Department of Health Sciences Universitat Oberta de Catalonia Barcelona Spain; ^5^ Department of Physiology, Faculty of Medicine Universitat de València Valencia Spain

**Keywords:** aerobic exercise, hyperhomocysteinemia, ketone medium‐chain triglyceride, mild cognitive impairment, vitamin B

Key Messages

- Mild cognitive impairment (MCI) is characterized by a decline in cognition and is associated with a higher risk of progression to dementia and Alzheimer's disease (AD).
- Considering the complexity and multifactorial aetiology of MCI, dementia and AD, there is a growing understanding that optimal preventive strategies are likely to require targeting several risk factors and mechanisms simultaneously.
- Recent evidence shows that interventions such as exercise, in particular aerobic exercise (AE), exogenous sources of ketones (namely ketogenic medium‐chain triglyceride [kMCT]) and supplementation with vitamins B_12_, B_6_ and folic acid may positively impact cognitive performance in MCI and AD.
- Although further studies might be necessary to understand the exact pathways through which this occurs, there are reasons to suspect that, in combination, these interventions could have a synergetic effect.
- In this paper, we hypothesize that a multicomponent cognitive therapy with ketogenic oral nutritional supplementation with vitamins B_12_, B_6_ and folic acid and AE could have a synergistic effect and help delay cognitive decline in patients with MCI. Further studies that include objective measurements of cognitive function are needed to confirm this hypothesis.

## 1 INTRODUCTION

Individuals with mild cognitive impairment (MCI) experience a decline in their cognitive abilities and are at an increased risk of developing Alzheimer's disease (AD) or other types of dementia.^1^ While some people with MCI remain stable or may return to normality, more than half experience progression to dementia within 5 years.^1^

There are multiple risk factors associated with MCI, categorized either as modifiable (e.g., comorbidities such as hypertension, type 2 diabetes [T2D], insulin resistance, suboptimal nutrient uptake, oxidative stress and malnutrition) or nonmodifiable factors (e.g., age, ethnicity, gender and genotype). Since the pathological process commences many years before the onset of AD, early identification and management of MCI is crucial for therapeutic interventions to help delay progression. Over the past decade, the potential of nutritional interventions to improve prognosis in MCI has been increasingly investigated.

In this paper, we focus on two important modifiable risk factors for MCI, namely reduced brain glucose uptake and increased plasma homocysteine (Hcy).^2^ Patients with MCI typically experience a nearly 10% decrease in their usual brain glucose metabolism, leading to a chronic brain energy shortage or a brain energy gap.^2^ However, brain ketone (acetoacetate) uptake and metabolism is not affected and can act as alternate energy substrate for the brain.^3^ As for Hcy, according to an 8‐year follow‐up study, the risk of AD has been shown to double with a plasma Hcy level >14 mol/L.^4^

We hereby present evidence‐based benefits of certain interventions used alone—such as exogenous ketone sources (viz. ketogenic medium‐chain triglyceride [kMCT]), vitamin B supplementation and aerobic exercise (AE)—and we explore the potential synergistic effects of combining these interventions.

## 2 BENEFITS OF SUPPLEMENTATION WITH KETOGENIC MEDIUM‐CHAIN TRIGLYCERIDES FOR COGNITION

As discussed earlier, it is now known that although brain glucose uptake is compromised in the context of cognitive impairment, ketone uptake is not affected and helps counter the brain energy deficit in such circumstances.^2^ Ketogenic interventions have been shown to positively impact brain function, and ketotherapeutics have been investigated in several preliminary studies for MCI and AD.^2^ These studies have established the role of ketogenic interventions in improving memory in individuals with MCI and demonstrated enhanced cognitive scores in participants with AD compared with placebo.^2^

Brain energy rescue is emerging as a potential strategy to reduce cognitive decline in MCI and AD.^3^ Brain glucose hypometabolism occurs *before the onset* of cognitive symptoms in those at increased risk of early‐ or late‐onset AD.^5^ Irrespective of reduced brain glucose uptake, brain ketone uptake remains normal in MCI and AD.^2^ Improved brain energy status through increased ketone uptake is positively correlated with improved cognitive outcomes.^5^

**FIGURE 1:**
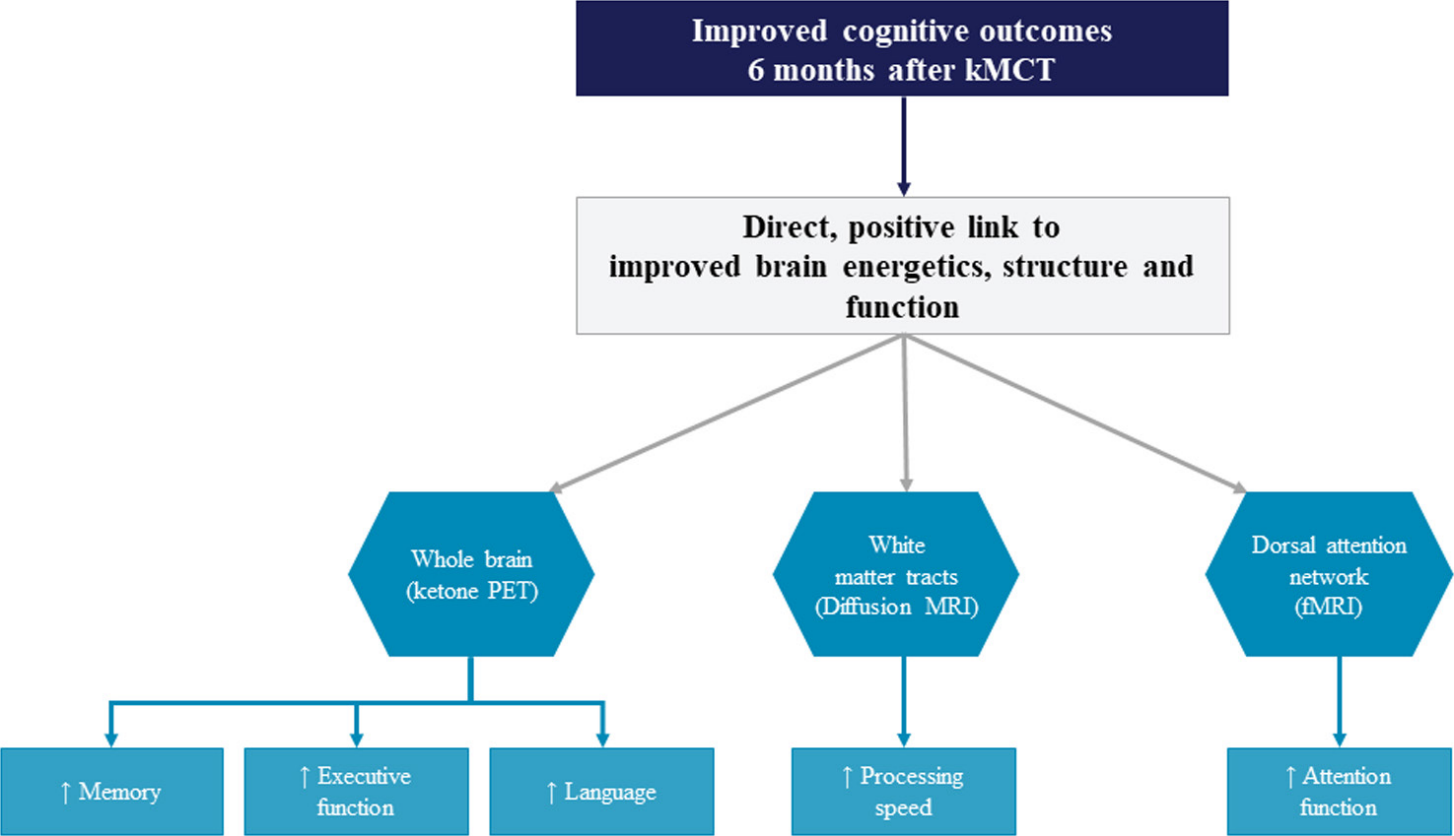
Improved cognitive outcomes 6 months after kMCT observed in the BENEFIC trial. Summary of cognitive outcomes that have improved 6 months after kMCT intervention starts in the BENEFIC trial. Figure elaborated by the authors based on results from Fortier et al.^5^ and Roy et al.^19^, ^20^. kMCT, ketogenic medium‐chain triglyceride; MRI, magnetic resonance imaging; PET, positron emission tomography

It has been shown that ketogenic supplements are a safe and simple way of enhancing plasma ketone levels and brain ketone uptake in AD.^2^ This observation was further extrapolated to MCI in the Brain ENErgy, Functional Imaging, and Cognition (BENEFIC) trial (NCT02551419), which explored the possibility of improving cognition in MCI when compromised brain glucose metabolism is countered with ketones through consumption of emulsified kMCT (BrainXpert Energy Complex drink).^5^ Patients with MCI (as per Peterson criteria) of all genders aged ≥55 years were randomized to two arms: those consuming 30 g/day of emulsified kMCT (*n* = 39) and those consuming the matching placebo (*n* = 44). The trial was conducted in two phases: Phase 1 demonstrated that consumption of kMCT enhanced brain ketone uptake and brain energy in participants on the kMCT compared with calorie‐matched placebo and that this increase was directly associated with better cognitive outcomes in the domains of memory, executive function and language. In phase 2 of the study, in addition to cognitive outcomes, participants underwent assessment of blood ketone response to the kMCT drink. The plasma ketone response was sustained after 6 months' consumption of kMCT and cognitive outcomes improved in the same cognitive domains 6 months after supplementation with kMCT as in phase 1.^5^ Moderate‐to‐large effect sizes (partial ƞ2 of 0.06–0.14) were achieved on 4 cognitive tests in the kMCT group—Free and Cued Recall Test (*p* = .047), Verbal Fluency Test (*p* = .024), Boston Naming Test increased by 1.1 (*p* = .033) and Trail Making Test (*p* = .017)—suggesting the clinical relevance of these cognitive improvements, especially in relation to memory, executive function and language. Global brain ketone uptake doubled for the kMCT arm and was directly correlated with elevated plasma ketones, while no change in brain ketone uptake occurred on placebo. The change in plasma total ketones was positively correlated with the change in several cognitive tests of episodic memory, executive function and language, with coefficients of correlation of *r* = +0.229 to +0.325 (*p* < .042–.0028). Uptake of ketones by the brain as a whole was directly related to improved episodic memory, verbal fluency and language^5^; processing speed improved as a function of ketone uptake in white matter,^19^ and attention improved as a function of ketone uptake and connectivity in the dorsal attention network^20^ (Figure 1). Therefore, the outcomes of this randomized controlled trial (RCT) provide support in favour of improved cognitive performance in MCI through ketone‐dependent brain energy rescue with kMCT. The treatment was also found to be safe and moderately well tolerated.^5^

## 3 IMPACT OF SUPPLEMENTATION WITH VITAMINS B_6_ , B_12_ AND FOLIC ACID ON THE REDUCTION OF HOMOCYSTEINE LEVEL

B‐vitamin deficiencies contribute to the pathogenesis of cognitive impairment through increased Hcy^6^ and are more likely observed in the elderly population.^6^ A systematic review assessed the influence of treatment with vitamins B_12_, B_6_ and/or folic acid (compared with baseline values) on Hcy levels in patients with MCI.^7^ All identified studies (8 studies, including a total of 1140 participants), irrespective of the duration of intervention, exhibited a statistically significant decrease in Hcy levels, ranging from 9.8 to 48.6% (mean decline of 31.9% in intervention arms and 0.7% increase in control arm) as early as a month after supplementation with vitamins B_6_, B_12_ and/or folic acid, compared with controls. The greatest decline in Hcy concentration—48.6%—was observed with a combination of vitamins B_6_, B_12_ and folic acid. These findings show that supplementation with vitamins B_6_, B_12_ and folic acid is an option to reduce Hcy levels in people with MCI and elevated plasma Hcy, thus mitigating the risks of high Hcy on neurological degeneration.

## 4 BENEFITS OF AEROBIC EXERCISE FOR COGNITION

Physical exercise, particularly combining aerobic and resistance training, has been postulated to have cognitive, mental health and life quality benefits in the older population, as well as in MCI and dementia, along with delaying the onset or progression of AD.^8^ An uncontrolled study showed that in mild AD, 3 months of moderate‐intensity AE produced a twofold increase in brain ketone uptake [(CMR_acac_, K_acac_ and dCMR_ket_), while brain glucose uptake remained unchanged.^9^ The increase in plasma and brain ketones achieved in this study was associated with some cognitive improvement, but the study was underpowered for definitive assessment of cognitive function. Other reports have also reported the contribution of AE to lowering total plasma Hcy and modulating blood Hcy levels in individuals with hyperhomocysteinemia (HHcy). These results suggest two different beneficial effects of AE in managing MCI.^10^ Interventions combining cognitive training and AE were also reported as potentially enhancing the intellectual and physical performance of adults with MCI, although further studies are needed to differentiate the contribution from each component.^11^

## 5 POTENTIAL SYNERGISTIC EFFECTS BETWEEN EXERCISE AND NUTRITIONAL SUPPLEMENTATION WITH KETOGENIC MEDIUM‐CHAIN TRIGLYCERIDE AND VITAMINS B_12_ , B_6_ AND FOLIC ACID

These observations point towards a synergistic effect between the various interventions that may alleviate cognitive impairment linked to AD.

Brain glucose deficit is countered by ketonemia, which is induced in response to decreased insulin to help fuel the brain's energy needs. Ketone transport into the brain remains unchanged during cognitively healthy ageing and directly corresponds with plasma ketone concentrations.^2^ However, insulin resistance and high blood insulin may dampen the availability and/or brain uptake of both glucose and ketones.^2^

Existing evidence supports the role of AE in increasing plasma ketones endogenously via mobilization of free fatty acids from adipose tissue of healthy adults, in turn facilitating brain ketone uptake in AD.^9^ While supplementation with dietary kMCT transiently induces mild ketosis,^9^ kMCT plus AE has been found to be more ketogenic compared with each intervention alone in older normoglycemic women, suggesting a synergistic effect of this combination on short‐term ketogenesis.^12^

Insulin resistance has been documented as an independent risk factor for the progression of abnormal glucose metabolism and increased risk of type 2 diabetes.^13^ Impaired glucose tolerance (IGT) is characterized by insulin resistance, and patients with IGT have higher Hcy levels compared with those with normal glucose tolerance (NGT).^14^ Of note, IGT is an intermediate stage between NGT and overt T2D and can transition to T2D; however, the responsible mechanisms remain unclear.^13^ HHcy is another factor associated with increased insulin resistance and hyperinsulinemia, thus promoting the development of insulin resistance diseases.^15^

A recent study demonstrated a prospective association between HHcy and the probability of having IGT and that Hcy might play a critical role in regulating glucose metabolism.^13^ There are a few possible mechanisms through which this might occur. Firstly, IGT might be a consequence of chronic exposure to severe insulin resistance and hyperinsulinemia, which is partly induced by HHcy, thus putting patients with HHcy at high risk for IGT.^13^ Secondly, oxidative stress might also be involved in mechanism linking HHcy to abnormal glucose metabolism.^13^ HHcy inhibits the production of glutathione (GSH), the major intracellular antioxidant, leading to high oxidative stress,^16^ which might also partially promote hyperglycaemia in the context of T2D pathology.^17^

Based on these findings, we propose that multiple parameters and factors such as brain glucose deficit, insulin resistance, IGT, HHcy and oxidative stress may have interlinked functional pathways that increase the risk of cognitive impairment in older people. We, therefore, hypothesize that combining supplementation of kMCT, vitamins B_12_, B_6_ and folic acid with AE could have a synergetic effect and help delay neurological degeneration in patients with MCI. We recommend that an RCT combining these elements should be designed and initiated to test this hypothesis.

## 6 CONCLUSION

**FIGURE 2:**
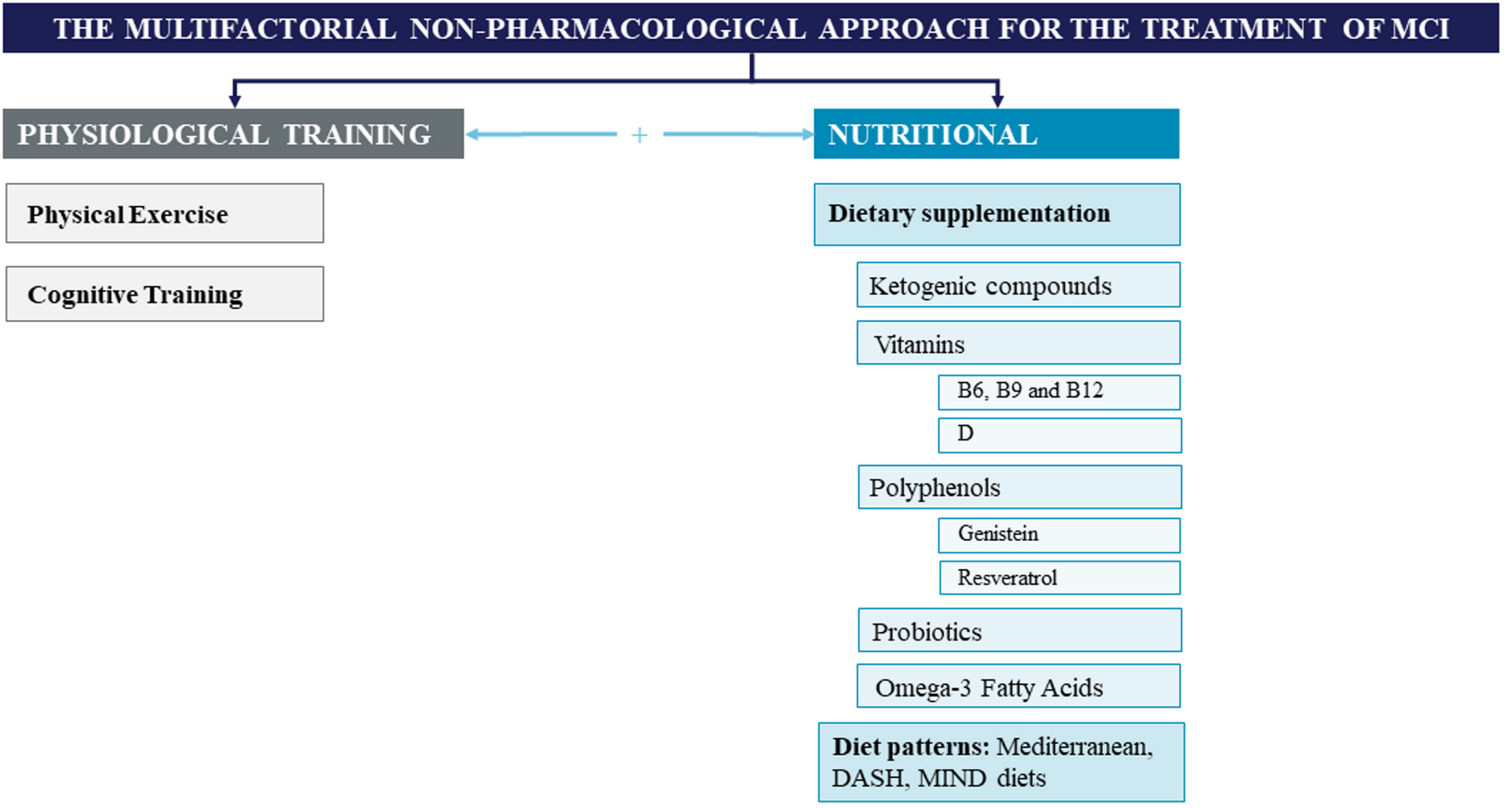
The Multifactorial non‐pharmacological approach for the treatment of MCI. In addition to the Nutritional component (Ketogenic compounds, Diet Patterns) and Physiological Training component (Physical Exercise and Cognitive Training) which we discuss in detail in this review and for which the evidence is increasingly solid, we have added some more speculative Nutritional components that are being actively researched at this time but which are not reviewed here (Vitamins, Polyphenols, Probiotics, Omega‐3 Fatty Acids). [Correction added on 01 June 2022, after first online publication: Some additional information added to figure legend in this current version.]

Although there are no approved pharmacologic options to treat MCI, mostly due to the challenges in demonstrating a beneficial impact on cognitive decline, there are modifiable risk factors that can be addressed through multifactorial non‐pharmacologic approaches (Figure 2). For example, innovative dietary supplementation with BrainXpert Energy Complex, made of encapsulated MCT, addresses some of these risk factors and was proven to have a positive effect on memory and cognitive function.^5^

Considering the complexity and multifactorial aetiology of MCI, dementia and AD, an optimal preventive strategy is likely to require targeting several risk factors and mechanisms simultaneously. Recent evidence shows that interventions such as AE, exogenous sources of ketones (viz. kMCT) and supplementation with vitamins B_12_, B_6_ and folic acid may positively impact cognitive performance in MCI or AD. Although further studies might be necessary to understand the exact pathways through which this occurs, these interventions could have a synergetic effect.

Currently, there are studies demonstrating synergistic effects of a combination of MCT and AE. A potential benefit of adding supplementation with vitamins B_12_, B_6_ and/or folic acid is postulated, due to its demonstrated ability of reducing homocysteine levels. As HHcy is an independent predictor of the risk for cognitive decline, this homocysteine‐lowering effect is expected to not only help prevent neurological degeneration in patients with MCI but also potentially help regulate glucose metabolism, which may facilitate the uptake of both brain fuels.

The promising approach of using nutritional interventions in combination with other interventions is steadily emerging and evolving in the management of MCI and prevention of AD. A good example is the 2‐year Finnish Geriatric Intervention Study to Prevent Cognitive Impairment and Disability (FINGER) trial, which used multidomain lifestyle intervention (diet, exercise, cognitive training and vascular risk monitoring) and demonstrated beneficial effects on cognition in older people.^18^

In summary, we hypothesize that a multicomponent cognitive therapy with ketogenic oral nutritional supplementation with vitamins B_12_, B_6_ and folic acid and AE could have a synergetic effect and help delay cognitive decline in patients with MCI. Further studies with objective measurements of cognitive function are needed to confirm this hypothesis.

## CONFLICT OF INTEREST

The authors of this manuscript have conflicts of interest to disclose. Stephen C. Cunnane has consulted for or received honoraria or test products for research from Nestlé Health Science, Bulletproof, Abbott, Cargill, Dr. Schär, Pro‐Diet, Cerecin and Abitec. His research is funded by NSERC, CIHR, CFI, FRQS, Université de Sherbrooke, Alzheimer Society of Canada and Alzheimer Association (USA). He is the founder and director of the consulting company, Senotec Ltd. Russell H. Swerdlow has consulted for or received honoraria for research from Nestlé Health Science. Marco Inzitari has consulted for or received honoraria for research from Nestlé Health Science. Gloria Olaso‐Gonzalez has consulted for or received honoraria for research from Nestlé Health Science. José Viña has consulted for or received honoraria for research from Nestlé Health Science. There are no other conflicts to report.
